# Transcriptomic and proteomic profiling of maize embryos exposed to camptothecin

**DOI:** 10.1186/1471-2229-11-91

**Published:** 2011-05-19

**Authors:** Nuria Sánchez-Pons, Sami Irar, Nora García-Muniz, Carlos M Vicient

**Affiliations:** 1 Department of Molecular Genetics, Centre for Research in Agricultural Genomics, Campus UAB, Edifici CRAG, Bellaterra (Cerdanyola del Vallés), 08193 Barcelona, Spain

## Abstract

**Background:**

Camptothecin is a plant alkaloid that specifically binds topoisomerase I, inhibiting its activity and inducing double stranded breaks in DNA, activating the cell responses to DNA damage and, in response to severe treatments, triggering cell death.

**Results:**

Comparative transcriptomic and proteomic analyses of maize embryos that had been exposed to camptothecin were conducted. Under the conditions used in this study, camptothecin did not induce extensive degradation in the genomic DNA but induced the transcription of genes involved in DNA repair and repressed genes involved in cell division. Camptothecin also affected the accumulation of several proteins involved in the stress response and induced the activity of certain calcium-dependent nucleases. We also detected changes in the expression and accumulation of different genes and proteins involved in post-translational regulatory processes.

**Conclusions:**

This study identified several genes and proteins that participate in DNA damage responses in plants. Some of them may be involved in general responses to stress, but others are candidate genes for specific involvement in DNA repair. Our results open a number of new avenues for researching and improving plant resistance to DNA injury.

## Background

Maintenance of genome stability is of critical importance for all organisms. Genomic DNA is continuously subject to many types of damage resulting from endogenous factors (production of reactive oxygen species, stalled replication forks, etc.) or the action of exogenous agents (radiation, naturally occurring radioisotopes, chemical mutagens such as heavy metals, etc.) [1]. Double-strand DNA breaks (DSBs) are one of the most serious forms of DNA damage, potentially causing chromosomal translocations and rearrangements [2]. In response to DSBs, cells initiate complex signalling pathways that activate DNA repair, cell-cycle arrest, and eventually cell death [3]. DSBs repair is mediated by two basic mechanisms: homologous recombination (HR) and non-homologous end joining (NHEJ) [4]. In HR, an intact copy of the damaged region (a sister chromatid, for example) acts as a template to repair the break. In NHEJ, DSBs are simply rejoined largely independently of the DNA sequence. Bacteria and yeast usually employ HR whereas mammals and plants usually use NHEJ.

In addition to the direct repair of DNA breaks, additional responses are activated during DNA-damage stress. For example, DNA damage in plant cells usually induces the accumulation of signal transduction intermediates such as nitric oxide, ROS or ethylene [5,6] and produces changes in the cytosolic-free Ca^2+ ^[7]. It also induces cell cycle arrest, the inhibition of DNA and RNA synthesis, and a rapid protein turnover via the proteasome [8,9]. Additional reported effects are a reduction in the photosynthesis-related proteins [10], the accumulation of protective proteins such as pathogenesis-related protein-1 [11], the accumulation of protecting pigments [12], an increase in the expression of senescence- and cell death-associated genes [13] and the activation of different cellular detoxification mechanisms [14]. The regulation of all these responses is complex and involves different levels of regulation, including the modulation of transcriptional activity [15], post-transcriptional mechanisms (RNA processing, RNA silencing, etc.) [16-18] and post-translational modifications (protein phosphorylation, ubiquitination, SUMOylation, etc.) [19]. These processes are based on signal transduction initiated by sensor proteins that recognise the damage in the DNA and activate the transducers, which send the signal to the effector proteins [20]. The network of transcriptional, post-transcriptional and post-translational modifications ensures temporally and spatially appropriate patterns of stress-responses.

DNA topoisomerase I (TOPI) regulates the topological state of DNA by cleaving and re-joining one DNA strand and allowing DNA relaxation [21]. TOPI activity is essential in dividing cells to release the torsion created by the progression of DNA replication forks. The presence of active TOPI is essential for embryo development in *Drosophila *and mouse [22]. In plants, TOPI plays a similar basic role and, for example, the disruption of the two TOPI encoding genes in *Arabidopsis thaliana *is lethal [23]. Camptothecin (CPT) is a plant alkaloid that specifically binds TOPI, stabilising the complexes formed between DNA and TOPI [24]. The collisions between the trapped TOPI-CPT complexes and the replication fork during DNA replication produce DSBs which induce DNA damage responses [25]. In consequence, actively dividing cells are much more sensitive to CPT than non-dividing cells, a property that has been exploited in the treatment of cancer [24]. However, non-dividing cells are also sensitive to CPT as collisions of the RNA polymerase machinery with the TOPI-CPT complexes, although less frequent, are also able to produce DSBs [26]. CPT-mediated TOPI-DNA complexes can be degraded via the 26S proteasome pathway so, at low CPT concentrations, cells can survive [27]. However, in actively dividing cells the high number of collisions may exceed the capacity of the cells to eliminate TOPI-DNA complexes and the DNA repair capability of the cells and, under these circumstances, cell death is initiated. CPT has a similar effect on TOPI in plant and animals. For example, CPT inhibits, *in vitro*, the activity of TOPI extracted from maize immature embryos [28], produces the abortion of shoots and roots in *Arabidopsis *[23], and induces cell death in tomato cell cultures [29].

In this study, we profiled proteins and genes whose expression is changed in immature maize embryos as a consequence of the DNA damage produced by CPT. Immature embryos contain a high proportion of actively dividing cells and, in consequence, are particularly sensitive to CPT. The combination of microarray and two-dimensional gel electrophoresis protein analysis allowed us to identify molecular events that are regulated during DNA repair responses in plants at different levels: transcriptional, post-transcriptional, translational and post-translational. We identified candidate genes and proteins which may be specifically involved in the DNA repair responses.

## Results

### Camptothecin induces DNA damage responses in maize immature embryos but not an extensive cell death process

Maize caryopses were collected 15 days after pollination and their dissected embryos incubated, in the dark, in culture medium with or without 50 μM camptothecin (CPT). After 8 days of culture, the germination rates of treated and untreated embryos were not significantly different (24% ± 5 in control and 20% ± 6 in treated embryos) and their morphological characteristics were similar.

**Figure 1:**
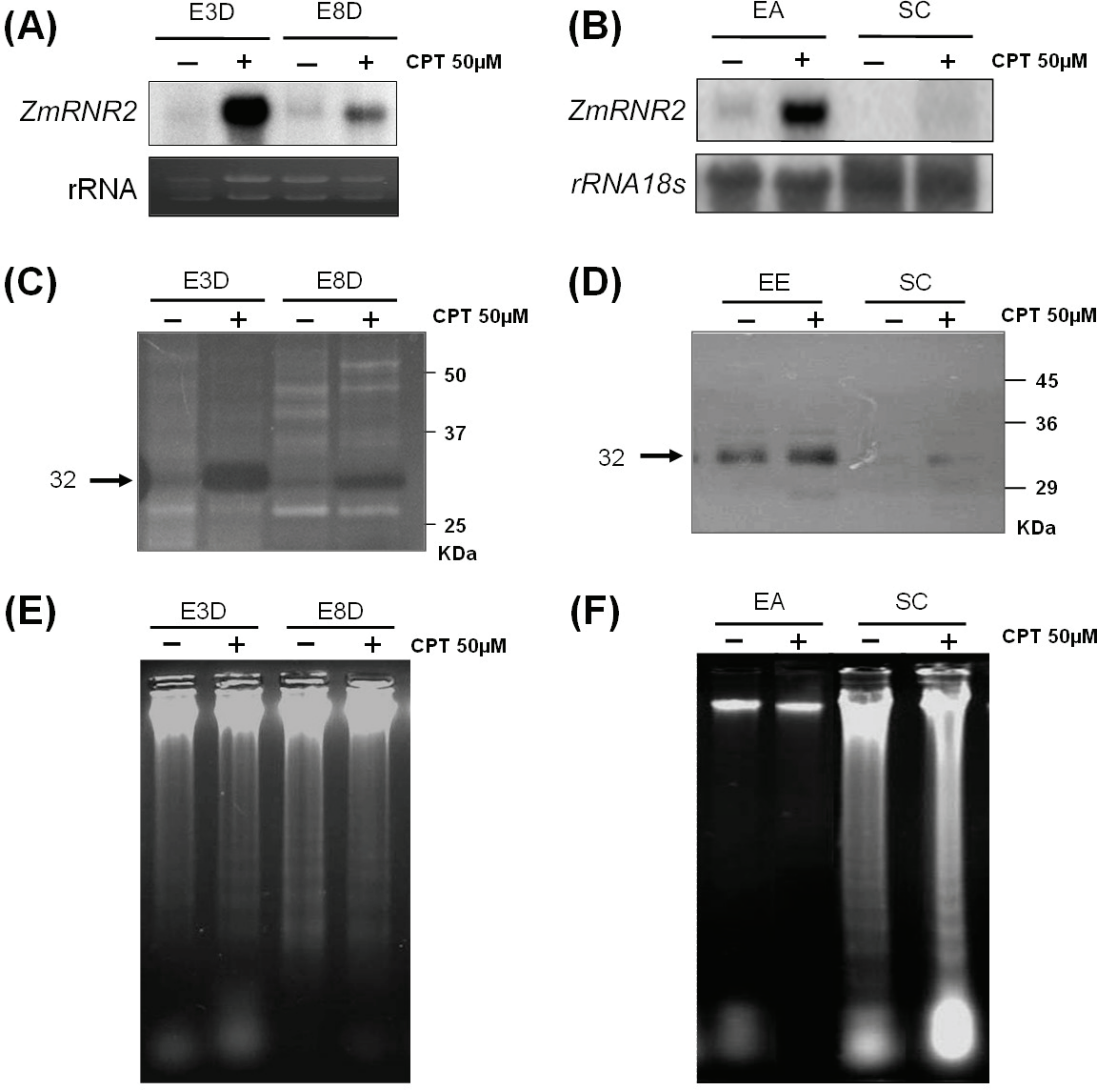
**CPT-induced DNA damage analysis**. **(A) **Northern blot of *ZmRNR2 *gene of immature maize embryos treated with 50 μM CPT for three (E3D) and eight (E8D) days. **(B) **Northern blot of *ZmRNR2 *gene of dissected embryo axis (EA) and scutellum (SC) of immature maize embryos treated with 50 μM CPT for three days. **(C) **In-gel nuclease activity assay of total protein extracts (10 μg) of immature maize embryos treated with 50 μM CPT for three (E3D) and eight (E8D) days. The nuclease activity is detected as a non-stained halo in a polyacrylamide gel containing DNA stained with ethidium bromide. The deduced weight of the proteins with nuclease activity is indicated on the left (kDa). **(D) **In-gel nuclease activity assay of total protein extracts (10 μg) of dissected embryo axis (EA) and scutellum (SC) of immature maize embryos treated with 50 μM CPT for three days. The deduced weights of the proteins with nuclease activity are indicated on the left (kDa). **(E) **Integrity of nuclear DNA (4 μg) of immature maize embryos treated with 50 μM CPT for three (E3D) and eight (E8D) days, assayed by electrophoresis on 1.5% agarose gels. **(F) **Integrity of nuclear DNA (4 μg) of dissected embryo axis (EA) and scutellum (SC) of immature maize embryos treated with 50 μM CPT for three days, assayed by electrophoresis on 1.5% agarose gels.

CPT is a DNA damaging agent that induces DNA repair responses [24], while ribonucleotide reductase (RNR) is an enzyme that provides dNTPs for DNA repair, with *RNR *genes being induced in response to DNA damage [30]. In order to check if, under our conditions, CPT is able to induce DNA repair responses in maize embryos, we used a maize gene *ZmRNR2 *probe encoding the ribonucleotide reductase, in northern blot hybridization (Figure 1A and 1B). There was a high level of accumulation of the *ZmRNR2 *mRNA after 3 days of CPT treatment and, although reduced, the accumulation was maintained after 8 days of treatment (Figure 1A). The induction of *ZmRNR2 *was much higher in the embryo axis than in the scutellum (Figure 1B).

Nucleases are involved in DNA damage responses [31] and in cell death [32]. In plants, cell death-related nucleases have been classified according to their cationic cofactors, as Ca^2+ ^or Zn^2+^-dependent [33]. The ability of CPT to induce nuclease activities in maize embryos was tested using in-gel nuclease activity assays (Figure 1C and 1D). An increase in the activity of a Ca^2+^-dependent nuclease of about 32 kDa was clearly evident after 3 days of CPT treatment using an assay buffer containing 1 mM CaCl_2_, being only slightly reduced after 8 days of treatment (Figure 1C), and was higher in the embryo axis compared to scutellum (Figure 1D). In contrast, no zinc-dependent nuclease activity was detected using 1, 2 or 5 mM Zn^2+ ^(results not shown).

The CPT-induced Ca^2+^-dependent nuclease could be involved in DNA repair but also in programmed cell death (PCD). PCD is usually characterised by inter-nucleosomal genomic DNA fragmentation, producing, after gel electrophoresis, a characteristic DNA ladder pattern [34]. The results of electrophoresis of genomic DNA extracted from treated embryos was not significantly different to that observed with untreated embryos, showing a certain DNA ladder (Figure 1E). The same analyses using DNA extracted separately from embryo axis and scutellum clearly show that the DNA ladder was only present in the scutellum sample (Figure 1F). Degradation in genomic DNA extracted from scutellum has been previously observed in maize [34]. Cells in the scutellum close to the embryo axis undergo PCD as a normal part of seed development and this may explain the observed DNA ladder [35]. Exposure to 50 μM CPT did not, however, produce a significant change in the DNA integrity in the embryo axis or scutellum. This suggests that the CPT-induced Ca^2+^-dependent nuclease is involved in DNA repair and not in cell death.

**Figure 2:**
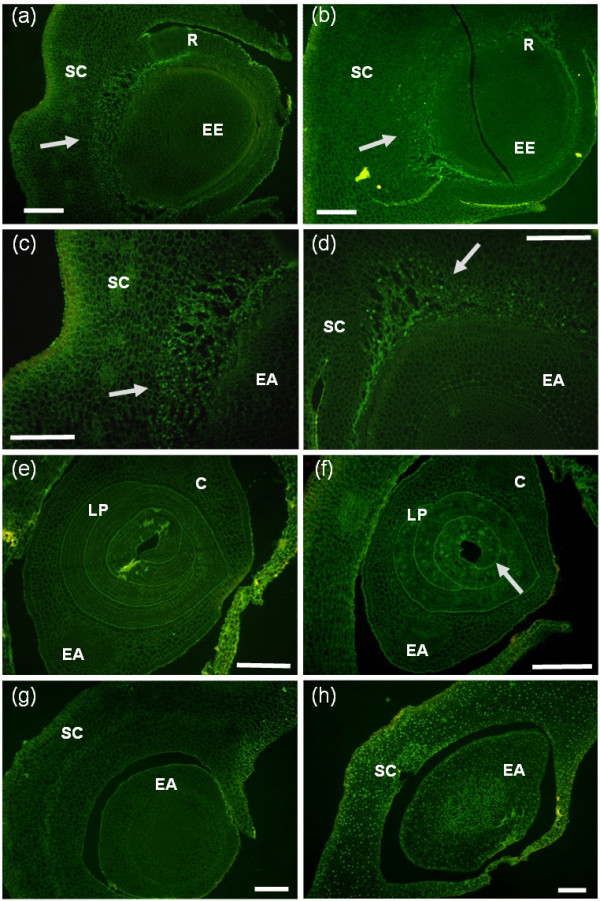
***In situ *detection of DNA fragmentation in histological sections of immature embryos treated with CPT**. TUNEL assay on histological sections of untreated embryos (a, c, and e) and embryos treated with 50 μM CPT for 3 days (b, d and f). The TdT enzyme was omitted in the negative control (g), and the positive control included a DNaseI incubation (h). Arrows indicate stained nuclei. SC, scutellum. EA, embryo axes. C, coleoptile. LP, leaf primordium. R, radicle. Scale bars: = 100 μm.

*In situ *detection of fragmented DNA (TUNEL), as a sensitive technique to detect the initial steps of genomic DNA degradation, was used to analyse the effects of CPT on maize embryo DNA integrity (Figure 2). In accordance with published data [35], untreated embryos only showed TUNEL positive nuclei in the scutellum, close to the embryo axis (Figures 2A and 2C). There was no increase in the number of positive nuclei in the scutellum of CPT treated embryos (Figure 2B and 2D). The embryo axis of untreated embryos did not show any TUNEL staining (Figure 2E). On the contrary, in CPT-treated embryos, some cells in the embryo axis showed TUNEL stained nuclei (Figure 2F). However, the proportion of cells with stained nuclei was not high, which may explain why we did not observe extensive genomic DNA degradation in gel electrophoresis.

These results indicate that, under the conditions used here, CPT induced DNA repair responses in maize embryos but not an extensive cell death process.

### Transcriptional responses to CPT-induced DNA damage

**Figure 3:**
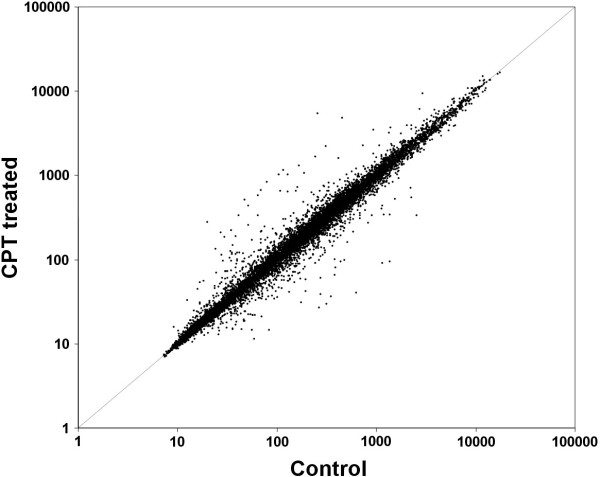
**A scatter plot of Affymetrix microarray analyses with mRNA from *Zea mays *immature embryos treated with camptothecin 50 μM for three days**.

**Figure 4:**
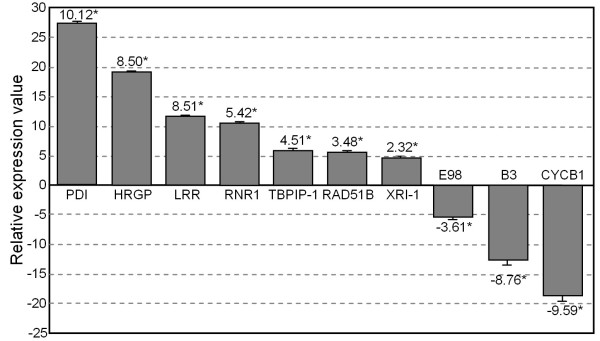
**Quantitative real time RT-PCR analysis of microarray results**. Real time RT-PCR of seven up-regulated and three down-regulated genes in response to camptothecin treatment. Relative expression values were normalised using actin as housekeeping gene. Induction values obtained in the microarray hybridization are indicated for each gene (*). The fold-change expression were calculated from three independent biological replicates; the biological replicates and standard errors are indicated (error bars). *PDI *(protein disulfide isomerase, U90944), HRGP (hydroxyproline-rich glycoprotein, BM073956), LRR (leucine rich repeats protein, CK370970), RNR1 (ribonucleotide reductase large subunit, BM079174), TBPIP1 (TBP-1 interacting protein, CA827618), RAD51B (AF079429) XRI-1 (X-ray induced gene 1, AY108750), CycB1 (cyclin IaZm, AI622454), B3 (B3 domain containing DNA binding protein, CO531505), E98 (HMG1/2 family transcription factor, AY104178).

A global picture of the changes in gene expression produced during CPT treatment was obtained using the Affymetrix™ GeneChip Maize Genome Array. In this experiment, control and 3-day CPT-treated embryos were compared (Figure 3). Ninety-three probe sets were found to have significantly increased or decreased signal in response to CPT, 39 up-regulated (Table 1) and 54 down-regulated (Table 2). The probe set corresponding to the *ZmRNR2 *gene, previously used as a control for DNA damage response, was among the up-regulated genes. A quantitative real-time RT-PCR approach was used to validate the expression of 10 genes identified as differentially expressed in the microarray analysis, including 7 up- and 3 down-regulated genes (Figure 4). Real-time PCR results were in very good agreement with the microarray data, although there were higher fold-changes using real time RT-PCR, which may be due to differences in the dynamic range and sensitivity of the two methods, as has been previously suggested [36].

**Table 1:** Genes up-regulated by CPT-induced DNA damage.

GO	Definition	GB# (EST) UniGene ID	Probe Set ID	**log**_**2**_**(R)**	FDR	p-value Fisher	p-value ANOVA	Arab.ortholog gene AGI code (BLAST core)
**Cell growth & division**								
	Mob1-like protein/cell cycle checkpoint regulation	BM334263 Zm.87024	Zm.15219.2.A1_a_at	1.8880	0.0189	0.0000	<0.001	At5g45550 (1e-100)
**Cell structure**								
	Putative hydroxyproline-rich glycoprotein	BM073956 Zm.1956	Zm.1956.1.S1_at	3.0867	0.0486	0.0004	0.003	At1g63830 (5e-87)
	Vegetative cell wall protein gp1 precursor	BM075217 Zm.2556	Zm.2556.1.A1_at	2.3581	0.0483	0.0004	0.003	At5g09530 (4e-55)
**Defense and stress responses**								
	Nucleoredoxin1/PDI-like protein	U90944 Zm.75215	Zm.411.1.A1_at	3.3395	0.0190	0.0000	<0.001	At1g60420 (0.0)
	Class III peroxidase precursor	BG874182 Zm.3932	Zm.14563.1.A1_s_at	2.8933	0.0411	0.0002	0.002	At1g68850 (1e-85)
	Acidic classI chitinase	L00973 Zm.93771	Zm.847.1.S1_at	2.5319	0.0190	0.0000	0.001	At3g12500 (7e-85)
	NEP1-interacting protein	BE051646 Zm.1499	Zm.1499.2.S1_a_at	2.0058	0.0190	0.0000	<0.001	At3g05880 (1e-19)
	Glutathione S-transferase GST 41	AF244706 Zm.81286	Zm.566.1.S1_at	1.3591	0.0486	0.0004	0.002	At3g09270 (7e-47)
	Glutathione S-transferase GST 36	AF244701 Zm.561	Zm.561.1.A1_at	1.0026	0.0486	0.0004	0.002	At3g09270 (1e-51)
**DNA replication, recombination and repair**								
	Ribonucleoside-diphosphate reductase small chain	AY105596 Zm.6802	Zm.14324.3.A1_x_at	2.6765	0.0425	0.0003	0.002	At3g27060 (1e-153)
	Ribonucleotide reductase R1 (large subunit)	BM079174 Zm.94425	Zm.5173.1.A1_at	2.4371	0.0205	0.0001	0.001	At2g21790 (1e-114)
	ZmRAD51B (AtRAD51)	AF079429 Zm.632	Zm.632.1.S1_at	1.7976	0.0271	0.0001	0.001	At5g20850 (1e-166)
	Putative DNA repair protein rhp16	AI665143 Zm.24329	ZmAffx.68.1.A1_at	1.6612	0.0041	0.0000	<0.001	At1g05120 (9e-73)
	Replication protein A2	AI691259 Zm.3800	Zm.3800.1.S1_at	1.5970	0.0189	0.0000	<0.001	At3g02920 (1e-44)
	Acetyltransferase, GNAT family protein	BM267811 Zm.9765	Zm.10129.1.A1_at	1.5045	0.0433	0.0003	0.002	At2g32030 (9e-47)
	Putative X-ray induced gene 1 (XRI-1)	AY108750 Zm.6271	Zm.6271.2.S1_a_at	1,.2268	0.0190	0.0000	<0.001	At5g48720 (7e-32)
**Energy**								
	NADH dehydrogenase I subunit N	AY108360 Zm.9290	Zm.9290.1.A1_at	1.5489	0.0483	0.0004	0.002	At5g58260 (1e-73)
	SC3 protein/Secretory carrier-associated membrane protein	CK826632 Zm.2391	Zm.2391.1.A1_at	1.3676	0.0282	0.0001	0.001	At1g61250 (1e-109)
**Metabolism**								
	Plastid ADP-glucose pyro-phosphorylase large subunit	BM379502 Zm.84929	Zm.12201.1.A1_at	2.7181	0.0486	0.0004	0.003	At5g19220 (0.0)
	Glucosyltransferase	CN844543 Zm.16431	Zm.16431.1.S1_at	1.4698	0.0483	0.0004	0.002	At3g16520 (3e-88)
**Protein processing**								
	Purple acid phosphatase 1	CF041723 Zm.3526	Zm.3526.1.S1_at	2.6090	0.0486	0.0004	0.003	At1g14700 (1e-117)
	Putative Tat binding prot.1 (TBP-1)-interact.prot.(TBPIP)	CA827618 Zm.13315	Zm.13315.1.S1_at	2.1725	0.0231	0.0001	0.001	At1g13330 (5e-65)
	PI31 Proteasome inhibitor-like protein	BM078279 Zm.6974	Zm.6974.1.A1_at	1.2613	0.0250	0.0001	0.001	At3g53970 (3e-37)
**Signal transduction andgene expression**								
	NAC domain-containing protein 77	BM379544 Zm.4179	Zm.4179.1.A1_at	2.3089	0.0098	0.0000	<0.001	At5g18270 (3e-61)
	NAC domain-containing protein 21/22	BM381180 Zm.76113	Zm.11843.1.A1_at	2.2599	0.0250	0.0001	0.001	At3g04060 (4e-21)
	Putative Rop family GTPase, ROP7 (AtROP9)	AY104576 Zm.14010	Zm.1279.1.S1_at	1.1436	0.0483	0.0004	0.002	At4g28950 (1e-100)
**Transposons**								
	Transposon protein Pong subclass	BM073216 Zm.2207	Zm.2207.1.A1_at	3.4310	0.0098	0.0000	<0.001	At2g13770 (2e-29)
**Unknown**								
	Unknown protein	CF637079 Zm.84375	Zm.3785.1.S1_at	4.4767	0.0041	0.0000	<0.001	At1g29640 (6e-13)
	Unknown protein	BM078256 Zm.84635	Zm.4210.1.S1_at	3.3151	0.0292	0.0001	0.001	At3g47070 (2e-06)
	Leucine-rich repeat, cysteine-containing protein	CK370970 Zm.98655	Zm.17789.1.A1_at	3.0884	0.0098	0.0000	<0.001	At2g06040 (4e-40)
	Unknown protein	CF974775 Zm.17071	Zm.17071.1.S1_at	2.5487	0.0143	0.0000	<0.001	At5g02220 (0.0)
	Unknown protein	BG840178 Zm.3570	Zm.3570.1.A1_at	2.0342	0.0141	0.0000	<0.001	At5g39530 (6e-08)
	Unknown protein	BM073017 Zm.2445	Zm.2445.1.A1_at	1.6888	0.0223	0.0001	0.001	Nd
	Histidine kinase-like ATPase superfamily	BQ485400 Zm.10451	Zm.10451.1.S1_at	1.5810	0.0189	0.0000	<0.001	At1g19100 (4e-04)
	Unknown protein	CO521239 Zm.19124	Zm.19124.1.A1_at	1.3036	0.0189	0.0000	<0.001	At5g35320 (2e-14)
	Unknown protein	AY106977 Zm.82291	Zm.2968.1.A1_at	1.2993	0.0483	0.0004	0.002	Nd
	Unknown protein	BG841197 Zm.61674	Zm.2192.1.A1_at	1.1864	0.0189	0.0000	<0.001	Nd
	Unknown protein	BQ538249 Zm.10551	Zm.10551.1.A1_at	1.0488	0.0438	0.0003	0.002	At1g31720 (6e-23)
	AAA-type ATPase/ATPase2	CK826796 Zm.94919	Zm.16211.1.S1_at	3.2748	0.0189	0.0000	<0.001	At3g28540 (0.0)

GB#, maize EST accession number in GenBank database. UniGene ID code; Probe set ID in Affymetrix chip; Affymetrix chip hybridization parameters: R ≥ 2.0 and false discovery rate (FDR) ≤ 0.05.

**Table 2:** Genes down-regulated by CPT-induced DNA damage.

GO	Definition	GB# (EST) UniGene ID	Probe Set ID	**log**_**2**_**(R)**	FDR	p-value Fisher	p-value ANOVA	Arab. ortholog gene AGI code (BLAST score)
**Cell growth and division**								
	SMC-like domain containing protein	AY111519 Zm.83602	Zm.6452.1.A1_at	-1.1538	0.0190	0.0000	<0.001	At3g20350 (1e-15)
	Shugoshin-1	EU967226 Zm.96142	Zm.6790.1.A1_at	-1.3230	0.0487	0.0005	0.003	At5g04320 (3e-14)
	Frizzy-like protein/WD-repeat cell cycle regulatory protein	BT036099 Zm.26408	Zm.4859.1.A1_at	-1.4030	0.0483	0.0004	0.002	At5g13840 (0.0)
	TPX2	AW231676 Zm.5454	Zm.5454.1.A1_at	-1.6822	0.0280	0.0001	0.001	At1g03780 (8e-23)
	Cyclin IIZm (CYCA1;1)	AI61499 Zm.3420	Zm.3420.1.A1_at	-1.7926	0.0433	0.0003	0.002	At1g44110 (1e-141)
	Cyclin IIIZm (CYCB2)	U10076 Zm.146	Zm.146.1.S1_at	-2.3331	0.0438	0.0003	0.002	At1g20610 (1e-119)
	Syntaxin-related protein KNOLLE	CD442886 Zm.96795	Zm.4845.2.S1_at	-3.0685	0.0205	0.0001	0.001	At1g08560 (2e-95)
	Cyclin IaZm (cyclin-B1;2/CYC1BAT)	AI622454 Zm.95231	Zm.4288.1.A1_at	-3.2616	0.0189	0.0000	<0.001	At5g06150 (1e-71)
	Patellin-5/SEC14 cytosolic factor-like	CF635836 Zm.6066	Zm.6066.1.A1_at	-1.7331	0.0486	0.0004	0.003	At1g30690 (1e-114)
**Cell structure**								
	Microtubule-associated protein RP/EB family member 3	AI586906 Zm.85067	Zm.6324.1.A1_at	-1.3018	0.0483	0.0004	0.002	At5g67270 (2e-28)
**Defense and stress responses**								
	Dirigent-like	EU964079 Zm.3141	Zm.3141.1.A1_at	-2.1655	0.0189	0.0000	<0.001	At5g42510 (2e-05)
	Dehydration-responsive protein RD22	U38791 Zm.265	Zm.265.1.A1_at	-1.6339	0.0411	0.0002	0.002	At5g25610 (2e-26)
**Membrane trafficking**								
	Vacuolar protein sorting-associated protein	BT023983 Zm.85507	Zm.12885.1.A1_at	-1.5344	0.0327	0.0002	0.001	At4g17140 (1e-157)
	Vacuolar protein sorting 13C protein-like	AY107427 Zm.66893	Zm.14047.1.S1_at	-1.5425	0.0190	0.0000	<0.001	At1g48090 (5e-31)
**Metabolism**								
	Glutamate dehydrogenase	D49475 Zm.44	Zm.44.1.S1_at	-1.0185	0.0410	0.0002	0.001	At5g18170 (0.0)
	Endoglucanase 1 precursor	CO527893 Zm.68006	Zm.4852.1.A1_at	-1.1952	0.0189	0.0000	<0.001	At1g70710 (0.0)
	Nucleotide pyrophosphatase/phosphodiesterase	CF646219 Zm.5570	Zm.5570.1.A1_at	-1.7576	0.0204	0.0000	<0.001	At5g50400 (1e-73)
**Protein processing**								
	Ubiquitin-conjugating enzyme	BG841009 Zm.93645	Zm.14028.3.A1_at	-2.6927	0.0190	0.0000	<0.001	At1g50490 (3e-65)
**Signal transduction and gene expression**								
	RRM-containing protein SEB-4	EU972664 Zm.95189	Zm.1141.2.A1_at	-1.1703	0.0486	0.0004	0.002	At1g78260 (1e-21)
	Myb-like DNA-binding domain containing protein	CF244262 Zm.95733	Zm.974.1.A1_at	-1.3440	0.0486	0.0004	0.002	At4g32730 (1e-26)
	Serine/arginine repetitive matrix protein 1	EU961539 Zm.85692	Zm.5999.1.A1_at	-2.2585	0.0389	0.0002	0.002	At3g24550 (2e-06)
	Transcriptional regulatory protein algP	EU952551 Zm.8612	Zm.1903.1.S1_at	-2.3186	0.0483	0.0004	0.003	At5g10430 (1e-13)
	Homeobox transcription factor KNOTTED1	BG266135 Zm.94710	Zm.6265.1.A1_at	-1.0252	0.0411	0.0002	0.001	At4g08150 (3e-98)
	VEF family protein/embryonic flower 2	AY232824 Zm.14303	Zm.14303.1.S1_at	-1.0796	0.0253	0.0001	0.001	At5g51230 (4e-74)
	Rough sheath1 (RS1)/Homeo-box protein knotted-1-like	L44133 Zm.95282	Zm.271.1.S1_at	-1.2708	0.0313	0.0001	0.001	At4g08150 (1e-103)
	Embryogenic callus protein 98b/HMG1/2-family protein	AY104178 Zm.67296	Zm.5524.1.S1_at	-1.8538	0.0433	0.0003	0.002	At4g23800 (1e-144)
	Growth-regulating factor 8-like (atGRF2)	AI619357 Zm.6781	Zm.6781.1.A1_at	-1.4776	0.0487	0.0004	0.003	At4g37740 (2e-31)
	B3 domain containing DNA binding protein	BT035134 Zm.18375	Zm.18375.1.S1_at	-3.1306	0.0190	0.0000	<0.001	At3g19184 (4e-35)
	Putative receptor protein kinase (ERECTA)	BE510364 Zm.7145	Zm.7145.1.A1_at	-1.0473	0.0233	0.0001	<0.001	At2g26330 (0.0)
	ATROPGEF7 Rho guanyl-nucleotide exchange factor	EU971244 Zm.85234	Zm.5362.1.A1_at	-1.2314	0.0229	0.0001	<0.001	At5g02010 (1e-163)
**Unknown**								
	Unknown protein	EU955329 Zm.7304	Zm.7304.1.A1_x_at	-1.0021	0.0324	0.0002	<0.001	At1g31335 (6e-19)
	Protein binding protein/ankyrin repeat family protein/hox1a	EU957633 Zm.94760	Zm.6575.1.A1_at	-1.0453	0.0189	0.0000	<0.001	At5g14230 (0.0)
	Unknown protein	EE289957 Zm.5555	Zm.5555.1.S1_at	-1.0503	0.0280	0.0001	0.001	At2g16270 (2e-06)
	Unknown protein	EU966578 Zm.1003	Zm.14948.1.A1_at	-1.0540	0.0486	0.0004	0.002	At5g44040 (8e-31)
	Zinc finger (C3HC4-type RING finger)-like protein	BT033558 Zm.85528	Zm.4717.1.A1_at	-1.1443	0.0244	0.0001	0.001	At5g60710 (3e-34)
	Lipid binding protein	BM380917 Zm.3374	Zm.3374.1.S1_at	-1.1558	0.0312	0.0001	0.001	At3g53980 (4e-33)
	Histidine kinase-like ATPases superfamily	BT062141 Zm.2931	Zm.2931.1.A1_at	-1.1903	0.0271	0.0001	0.001	At5g50780 (0.0)
	Unknown protein	AY108387 Zm.1851	Zm.1851.1.A1_at	-1.3130	0.0486	0.0004	0.002	At3g15560 (4e-06)
	Alpha-amylase inhibitor, lipid transfer & seed storage protein	EU966009 Zm.84843	Zm.1477.1.S1_at	-1.3289	0.0233	0.0001	0.001	At1g62790 (1e-20)
	Uncharacterized plant-specific domain TIGR01568 protein	EU964402 Zm.4518	Zm.4518.1.A1_at	-1.3515	0.0271	0.0001	0.001	At1g31810 (3e-12)
	Unknown protein	CB331155 Zm.14601	Zm.14601.1.A1_at	-1.4546	0.0424	0.0003	0.002	At1g65710 (7e-30)
	Putative mitochondrial glycoprotein	CN071241 Zm.10190	Zm.10190.1.S1_at	-1.5186	0.0313	0.0001	0.001	At3g55605 (2e-35)
	Unknown protein	EU953101 Zm.7304	Zm.7304.2.S1_x_at	-1.7155	0.0190	0.0000	0.001	At1g31335 (2e-18)
	Glycin-rich protein 3 (ZmGrp3)	Y07781 Zm.81016	Zm.106.1.A1_at	-1.8099	0.0478	0.0003	0.002	At5g46730 (3e-48)
	Unknown protein/Armadillo-type fold	CO523236 Zm.17093	Zm.17093.1.S1_at	-2.0104	0.0487	0.0004	0.003	At4g15830 (1e-65)
	Unknown protein	EU949556 Zm.6891	Zm.6891.1.S1_at	-2.1106	0.0205	0.0001	0.001	At1g16630 (9e-14)
	Unknown protein	EU952572 Zm.12124	Zm.12124.1.A1_at	-2.1345	0.0313	0.0001	0.001	At1g16610 (3e-04)
	Unknown protein	BT033638 Zm.74351	Zm.5168.1.A1_at	-2.1775	0.0098	0.0000	<0.001	At2g30820 (2e-33)
	Unknown protein	EU947738 Zm.13423	Zm.13423.1.A1_at	-2.4249	0.0173	0.0000	<0.001	nd
	Unknown protein	AI395973 Zm.6726	Zm.6726.1.S1_x_at	-2.5460	0.0419	0.0003	<0.001	At5g16250 (1e-48)
	Unknown protein	EU966355 Zm.4821	Zm.4821.1.S1_at	-2.5761	0.0313	0.0001	0.001	At2g29210 (4e-05)
	Glycine rich protein 3	CK371522 Zm.98965	Zm.17547.1.S1_at	-2.7476	0.0233	0.0001	0.001	At5g46730 (2e-50)
	Unknown protein	AY112394 Zm.6726	Zm.6726.2.A1_at	-3.0188	0.0189	0.0000	0.002	At5g16250 (3e-41)
	Unknown protein	CF627668 Zm.17534	Zm.17534.2.S1_at	-3.7711	0.0271	0.0001	0.001	At5g36710 (1e-38)

GB#, maize EST accession number in GenBank database. UniGene ID code; Probe set ID in Affymetrix chip; Affymetrix chip hybridization parameters: R ≤ 0.5 and FDR ≤ 0.05.

**Figure 5:**
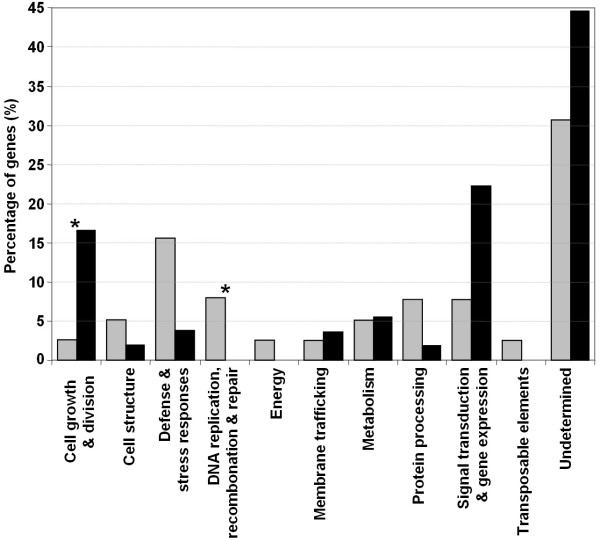
**Functional categories of differentially expressed genes**. Functional categories of the camptothecin induced (grey bars) and repressed (black bars) genes identified by microarray analysis. The functional categories significantly overrepresented between the induced or repressed genes are indicated by an asterisk (Fisher's Exact Test, p ≤ 0.05).

The molecular roles of many of the altered genes remain unknown (31% of the up-regulated and 44% of the down-regulated). These genes may be involved in the control and/or execution of DNA damage responses (Figure 5). DNA replication, recombination and repair (18%) and defense and stress responses (15%) were the two most abundant functional categories among the up-regulated genes. Among down-regulated genes, the two most abundantly represented categories were signal transduction and gene expression (22%) and cell growth and division (17%). The functional category of DNA replication, recombination and repair was significantly more represented among the induced genes while the cell growth and division category was significantly more represented among the repressed genes (Figure 5).

CPT treatment induced the expression of genes involved in DNA repair and DNA damage responses as, for example:

- Two subunits of the ribonucleotide reductase: involved in the DNA repair processes [30].

- *RAD51*: encodes a protein required for meiosis and HR repair [37]. Maize mutants in two *RAD51 *maize genes are hypersensitive to radiation [38]. The Arabidopsis gene *AtRAD51a *is transcriptionally up-regulated by DSB-inducing agents and seems to be required for HR repair after bleomycin treatment [39].

- *Rpa2*: encodes a protein that is part of a heterotrimeric protein complex that specifically binds single-stranded DNA (ssDNA) and plays multiple roles in DNA metabolism, including DNA repair and recombination [40]. *RPA *genes are transcriptionally induced in *Aspergillus nidulans *exposed to CPT [41].

- *TBPIP1*: encodes a protein involved in chromosome pairing and segregation [42]. In humans, TBPIP1 enhances the strand exchange mediated by RAD51 [43]. In Arabidopsis, the *TBPIP1 *gene is transcriptionally induced by DNA damage [44].

*- XRI-1*: encodes a protein essential for meiosis and that plays a role during HR in Arabidopsis [45]. This gene is highly and rapidly transcriptionally induced by X-ray radiation and is also highly induced by other DSBs-inducer agents [44]. The encoded protein is probably part of the meiotic recombination complex MND1/AHP2, which collaborates with RAD51 in the DNA strand invasion during recombination [46].

- Acetyltransferase, GNAT family protein: some yeast GNAT family members are involved in DSBs repair [47].

- *Rph16*: encodes a protein similar to RAD16 and is involved in the nucleotide excision repair of UV damage [48].

CPT treatment repressed the expression of genes involved in cell cycle, cell division and cell growth (Table 2). For example:

- Three cyclins: IaZm, IIZm and IIIZm.

*- Shugosin-1*: encodes a protein involved in the maintenance of centromeric cohesion of sister chromatids during meiosis and mitosis. Depletion of the human *Sgo1 *gene produces mitotic cell cycle arrest [49].

- *TPX2*: encodes a protein necessary for mitotic fuse formation in vertebrates [50]. The inhibition of the Arabidopsis *TPX2 *gene blocks mitosis [51].

- *Knolle*: encodes a syntaxin-like protein that acts during cytokinesis vesicle fusion and mediates cell-plate formation [52]. *Knolle *expression is repressed by gamma radiation in Arabidopsis [15].

- *Patellin-5*: patellins are involved in vesicle trafficking events. The Arabidopsis patellin PATL1 has been associated with the formation of the cell-plate during cytokinesis [53].

- *Knotted1 *(*Kn1*): encodes a homeo-domain protein involved in the regulation of leaf cell development [54].

- Microtubule-associated protein RP/EB family member 3: encodes a protein that binds to the end of the microtubules and is important in maintaining the structure of the mitotic spindle [55].

- *Growth regulating factor 8-like*: encodes a protein involved in leaf and cotyledon growth in Arabidopsis [56].

- *Rough sheath1: *encodes a protein involved in cell differentiation [57].

- Frizzy-like protein/WD-repeat cell cycle regulatory protein: encodes a protein similar to the tomato CCS52B that probably is involved in cell-cycle control during mitosis [58].

### Alterations in maize embryo proteome in response to CPT

**Figure 6:**
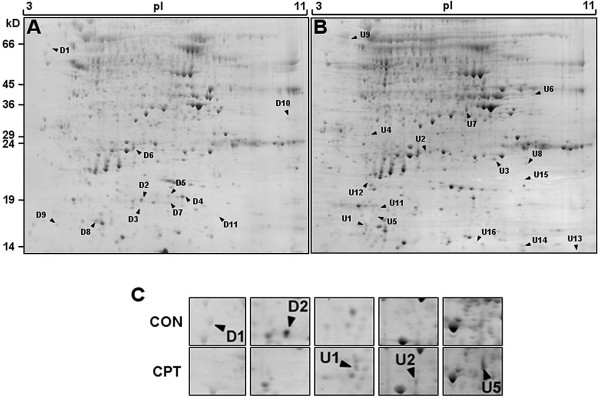
**Comparative proteomic analysis of CPT-induced DNA damage in maize immature embryos**. 2-D gel electrophoresis of untreated **(A) **and camptothecin-treated **(B) **maize embryos showing the localisation of the spots identified by mass spectrometry. The pH gradient, 3 to 11, is indicated on the top axis and molecular masses on the left. Spots that were excised and identified by mass spectrometry are labeled with spot ID numbers [U1-16, spots up-accumulated by CPT treatment; D1-14, spots down-accumulated by CPT treatment] listed in Table 3. **(C) **Portions of 2-D gels showing spots (arrows) that were differentially abundant between untreated (CON) and treated (CPT) embryos.

Equal amounts of total protein extracted from control and from CPT-treated maize embryos were fractionated using 2-D gel electrophoresis (Figure 6A and 6B). At least three-fold increase/decrease and t-test p < 0.05 were used as the criteria to select differentially accumulated polypeptides. In response to CPT treatment, 455 spots showed quantitative or qualitative (presence/absence) variations between the two gels, with the intensity decreasing in 169 and increasing in 286. Some examples of up- or down-accumulated spots are shown in figure 6C. Forty-three of the spots with significant differential expression on gels were chosen for identification by MS/MS mass spectrometry. Interpretable MS/MS spectra were obtained for 31 spots. The location of these in the gels is shown in Figures 6A and 6B.

The identified proteins belong to a variety of functional categories (Table 3). For example, CPT alters the accumulation of two enzymes involved in glycolytic metabolism: glyceraldehyde-3-phosphate dehydrogenase (GAPDH) and triosephosphate isomerase 1. Interestingly, in human neuronal cells, CPT also produces changes in the accumulation of GAPDH [59]. In plants, the accumulation of both proteins has been described in response to different types of stress [60-64].

**Table 3:** List of the spots/proteins identified by mass spectrometry.

GO	Spot ID	Protein (Species)	GB	Score	% Cv	Mw (kD) (Exp/Theo)	pI (Exp/The)	Pep-tides	CPT/CON
**Stress responses**									
	**U1**	Chloroplast Cu/Zn superoxide dismutase (maize)	NP001108127	128	24	17.51/20.99	4.71/5.45	2	CPT
	**U2**	Cytosolic ascorbate peroxidase (maize)	ACG38188	114	11	26.76/27.46	5.35/5.55	3	3.25
	**U3**	Glutathione S-transferase 19 (maize)	AAG34827	78	12	24.60/25.40	6.37/7.68	2	CPT
**Defense**									
	**D1**	Pathogenesis-related protein 1 (maize)	ACG29538	150	34	17.10/17.07	4.98/5.39	3	CON
	**D2**	Bet v I allergen (maize)	AY754698	66	24	18.30/17.07	3.80/4.68	3	CON
**Protein degradation**									
	**D3**	26S proteasome non-ATPase regulatory subunit 4 (maize)	ACG37494	138	6	60.32/42.4	3.54/4.46	2	CON
	**U4**	26S proteasome non-ATPase regulatory subunit 8 (maize)	ACG40420	138	14	29.87/31.05	4.70/5.03	3	CPT
**Protein synthesis**									
	**D4**	Eukaryotic translation initiation factor eIF-5A (maize)	CAA69225	197	70	20.01/17.70	5.38/5.61	7	CON
	**D5**	Eukaryotic translation initiation factor eIF-6 (maize)	ACG33598	92	6	26.93/27.01	3.95/4.64	1	0.46
**DNA replication, recombination & repair**									
	**D6**	Histone H2B (maize)	CAA40564	48	12	19.55/16.41	5.75/10.00	2	CON
**RNA metabolism**									
	**D7**	RraA (rice)	NP916709	101	26	19.01/18.06	5.35/6.12	4	0.5
	**U5**	RraA (maize)	ACG30537	307	39	18.58/18.19	4.95/5.33	6	CPT
	**D8**	DEAD-family RNA helicase (rice)	NP921002	128	30	27.72/26.60	6.46/6.82	5	CON
	**U6**	Glycine-rich RNA-binding protein 8 (maize)	ACG35695	70	8	23.60/21.38	7.00/7.63	2	CPT
	**D9**	RNA recognition motif containing protein (maize)	ACN31194	150	15	32.33/30.23	9.01/8.57	3	0.87
**Metabolism**									
	**D10**	Cytosolic GAPDH (maize)	CAA51676	70	4	20.19/36.63	5.88/6.46	1	CON
	**U7**	Glyceraldehyde-3-phosphate dehydrogenase (Arabidopsis)	AAK15554	74	14	40.95/44.97	7.24/8.75	4	3.23
	**D11**	Glyceraldehyde-3-phosphate dehydrogenase (Arabidopsis)	JQ1287	78	4	20.19/37.07	5.88/6.34	4	CON
	**D12**	Triosephosphate isomerase 1 (maize)	AAB81110	139	40	25.94/27.24	5.34/5.52	8	CON
**Signalling**									
	**U8**	Protein-G β-subunit (rice)	NP916988	100	21	36.34/36.66	5.86/5.97	4	2.84
**Endomembrane trafficking**									
	**D13**	Syntaxin 6 (rice)	NP001065843	121	42	32.32/24.10	6.70/5.56	7	CON
**Energy**									
	**U9**	Mitochondrial ATP synthase precursor (maize)	ACG38121	84	11	29.87/25.57	4.7/7.71	2	CPT
**Embryo and seed storage**									
	**U10**	Vicilin (rice)	CAA41810	86	15	14.12/65.45	5.00/6.63	5	CPT
	**U11**	Vicilin (rice)	CAA41810	132	21	19.48/65.45	4.99/6.63	7	CPT
	**U12**	Vicilin (rice)	CAA41810	110	8	22.12/65.45	4.77/6.63	3	CPT
	**U13**	Vicilin (rice)	CAA41810	75	2	14.2/66.63	9.3/6.23	1	CPT
	**U14**	globulin 2 (maize)	1802402A	64	3	15.03/50.23	6.03/6.16	1	CPT
	**D14**	globulin 2 (maize)	1802402A	66	2	17.93/50.23	6.64/6.16	1	CON
	**U15**	globulin 2 (maize)	1802402A	90	16	21.94/50.23	6.95/6.16	6	2.37
**Unknown**									
	**D15**	cupin domain containing protein (rice)	EAY88907	68	3	20.19/68.6	5.21/5.74	1	CON
	**U16**	r40c1 protein (rice)	ACF87898	71	3	19.6/38.77	6.2/6.3	1	CPT

U, proteins up-regulated by CPT. D, proteins down-regulated by CPT. GB, protein accession number in GenBank. Cv, coverage percentage. Mw/pI, theoretical and experimental molecular weight and isoelectric point. Peptides, tryptic peptides number. Changes in the relative spot volume are represented CPT/CON ratio of the mean from three independent replicas. CPT means presence only in treated samples, and CON presence only in control samples. The proteins were classified according to GO functional categories.

Some of the identified proteins are involved in antioxidant responses. Antioxidant activity protects against ROS accumulation, which can be produced by a variety of stresses, including DNA damage [65]. In mammals, CPT induces the accumulation of antioxidant enzymes in the nucleus [66]. The accumulation of two proteins involved in pathogenesis responses, PR1 and Betv1, was observed in response to CPT. They are also induced by abiotic stresses such as heavy metals [67] and UV radiation [68].

The accumulation of at least two 26s proteasome regulatory subunits is altered in response to CPT, one increased and the other reduced. Interestingly, CPT-TOPI-DNA complexes may be degraded by the ubiquitin-dependent pathway [27].

We observed changes in the accumulation of several proteins involved in RNA metabolism or RNA binding proteins:

- RraA: an RNaseE inhibitor which may be involved in the degradosome complex in *E. coli *[69,70]. Regulation of RraA by DNA damage stress could be related to changes in the regulation of RNA homeostasis.

- DEAD-family RNA helicase: DEAD-RNA helicases act in RNA metabolism promoting either RNA synthesis or decay [71]. Some have been associated with abiotic stress [72].

- Glycine-rich RNA-binding protein 8: some glycine-rich RBPs in Arabidopsis (GR-RBPs) are significantly induced by cold, drought and salinity, whereas others are repressed by other sources of stresses [73].

- RNA recognition motif-containing (RRM) protein: RRM-containing proteins are involved in most post-transcriptional gene expression processes (i.e. mRNA and rRNA processing, RNA export and stability) [74].

We also observed an increase in the accumulation of two spots corresponding to eukaryotic elongation factors. Experiments in yeast and mammals demonstrate that translation initiation factor 5A (eIF5A) is actually involved in mRNA nucleus-cytoplasm export and not translation, specifically regulating genes involved in cell growth and proliferation, and in cell death [75]. In Arabidopsis, *AteIF5A/AtFBR12 *(At1g26630) promotes PCD associated with the hypersensitive pathogen response [76], and *AteIEF5A-1 *(At1g13950) has been associated with PCD during xylogenesis [77]. Thus, regulation of eiF5A by CPT suggests it is involved in cell cycle and PCD regulation.

### Lack of correlation between CPT-induced changes in protein abundance and changes in mRNA accumulation

**Figure 7:**
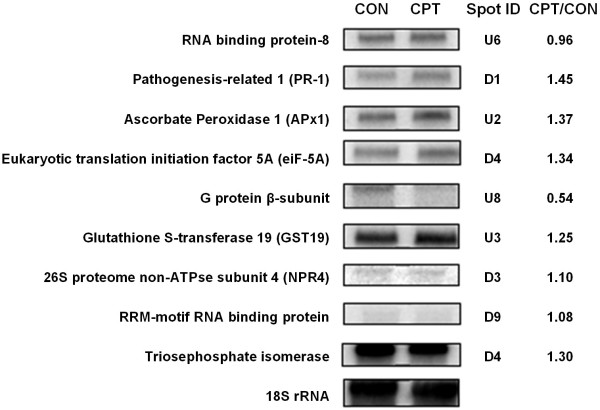
**Northern blot analysis of genes encoding proteins identified by comparative proteomic analysis**. Northern blot analysis (20 μg of total RNA per sample) showing expression of nine selected genes coding for proteins identified by comparative proteomic analysis in immature maize embryos treated with camptothecin 50 μM: glycine-rich RNA-binding protein 8, EU963577; pathogenesis-related protein 1, AY103811; ascorbate peroxidase 1, NM_001112030; eukaryotic translation initiation factor 5A, AY103556; guanine nucleotide-binding protein beta subunit-like, EU952736; glutathione S-transferase 19, AF244684; 26S proteasome non-ATPase subunit 4, BT018579; putative RNA-binding protein (RRM superfamily), BT065318; triosephosphate isomerase 1, AY103629]. Hybridization of the 18S ribosomal RNA was used as loading control. The corresponding protein spot ID and the relative fold-change (CPT/CON) normalised by 18S rRNA are indicated.

This study provided data on the most differentially expressed genes in control and CPT-treated embryos, and the most differentially accumulated proteins, allowing us to compare the datasets. The genes encoding 24 of the 31 identified proteins are represented in the microarray, but there was no significant change in expression in response to CPT (Table 4). This was confirmed by northern blot hybridizations using probes corresponding to nine of these genes, with no significant differences in the hybridization intensities observed (Figure 7).

**Table 4:** GeneChip array data analysis of the maize genes encoding the proteins identified by mass spectrometry.

Spot ID	Gene	TBLASTN		Affymetrix chip data			
		GB#	Score	Probe Set ID	**Log**_**2 **_**(R)**	p-value	FDR
**U1**	**Chloroplast Cu/Zn superoxide dismutase**						
		NM_001114655	0	Zm.9031.1.S1_at	-0.19	0.18	0.74
**U2**	**Cytosolic ascorbate peroxidase 1**						
		EU966070	1e-169	Zm.3633.8.A1_at	0.18	0.58	0.96
		n.d.	1e-163	Zm.3633.6.S1_at	0.23	0.42	0.92
		n.d.	1e-150	Zm.3633.8.S1_x_at	0.28	0.43	0.93
		n.d.	1e-144	Zm.3633.2.S1_x_at	0.01	0.93	1.00
		n.d.	1e-138	Zm.3633.3.S1_x_at	0.07	0.74	0.98
**U3**	**Glutathione S-transferase GST19**						
		AF244684	0	Zm.548.1.S1_at	0.12	0.59	0.96
**D3**	**26S proteasome non-ATPase regulatory subunit 4**						
		EU965376	0.00	Zm.5851.1.A1_at	0.15	0.12	0.64
		n.d.	1e-32	Zm.7644.2.S1_x_at	0.18	0.29	0.85
**U2**	**26S proteasome non-ATPase regulatory subunit 8**						
		EU968302	1e-154	Zm.6666.1.S1_at	-0.11	0.52	0.95
		n.d.	4e-97	Zm.6666.2.S1_at	-0.02	0.91	1.00
**D4**	**Eukaryotic translation initiation factor eIF-5A**						
		Y07920	0.00	Zm.1314.1.A1_at	0.10	0.29	0.85
		n.d.	0.00	Zm.3545.1.A1_at	0.14	0.14	0.68
**D5**	**Eukaryotic translation initiation factor eIF-6**						
		EU961480	0.00	Zm.7096.1.A1_at	0.13	0.50	0.94
**D10**	**Glyceraldehyde-3-phosphate dehydrogenase**						
		X07156	0.00	Zm.3765.1.S1_s_at	0.08	0.52	0.95
		n.d.	0.00	AFFX-Zm_Gapdh_M_f_at	0.11	0.53	0.95
		n.d.	0.00	AFFX-Zm_Gapdh_5_f_at	0.08	0.78	0.99
		n.d.	1e-118	Zm.16502.1.S1_at	0.13	0.51	0.95
		n.d.	2e-99	AFFX-Zm_Gapdh_3_s_at	0.10	0.33	0.87
		n.d.	6e-96	AFFX-Zm-gapdh-M_s_at	0.27	0.65	0.97
**U7**	**Glyceraldehyde-3-phosphate dehydrogenase**						
		AF348583	1e-19	Zm.8992.2.A1_a_at	-0.16	0.62	0.96
**D11**	**Glyceraldehyde-3-phosphate dehydrogenase**						
		AF348583	5e-10	AFFX-Zm_Gapdh_M_f_at	0.11	0.53	0.95
**D12**	**Triosaphosphate isomerase 1**						
		AH005585	1e-102	Zm.3889.1.A1_at	0.44	0.01	0.23
		n.d.	6e-67	Zm.3889.6.S1_at	0.15	0.50	0.94
**D6**	**Histone H2B**						
		X57312	0.00	Zm.16065.1.S1_at	0.30	0.20	0.76
		n.d.	1e-134	Zm.14497.4.A1_at	0.36	0.15	0.70
		n.d.	1e-124	Zm.14497.1.A1_at	0.22	0.71	0.98
		n.d.	1e-115	Zm.15914.3.A1_s_at	0.63	0.19	0.74
**D7**	**Regulator of RNAse activity A**						
		NM_191820	6e-94	Zm.12138.1.S1_at	0.06	0.85	0.99
		n.d.	1e-61	Zm.13462.1.A1_at	0.20	0.04	0.43
**U5**	**Regulator of RNAse activity A**						
		EU958419	0.00	Zm.12138.1.S1_at	0.06	0.85	0.99
		n.d.	3e-32	Zm.13462.1.A1_at	0.20	0.04	0.43
**D6**	**DEAD-family RNA helicase**						
		BT066085	0.00	Zm.13462.1.A1_at	0.20	0.04	0.43
		n.d.	1e-37	Zm.12138.1.S1_at	0.06	0.85	0.99
**U6**	**Glycine-rich RNA-binding protein 8**						
		EU963577	1e-119	Zm.13944.7.A1_a_at	0.07	0.67	0.97
		n.d.	2e-73	Zm.13944.6.S1_a_at	-0.15	0.69	0.98
**D9**	**RNA recognition motif containing protein**						
		BT065318	0	Zm.6941.1.A1_at	-0.17	0.07	0.51
		n.d.	1e-133	Zm.18107.1.S1_s_at	-0.07	0.61	0.96
		n.d.	1e-97	ZmAffx.915.1.S1_at	0.00	0.99	1.00
**D1**	**Pathogenesis-related protein 1**						
		EU957420	0.00	Zm.1967.1.A1_at	0.97	0.18	0.73
**D2**	**Bet v I allergen**						
		AY754698	1e-164	Zm.7135.1.S1_at	0.26	0.19	0.75
**U8**	**G-protein β-subunit**						
		NM_192099	3e-45	Zm.6045.7.A1_a_at	-0.10	0.40	0.91
**D13**	**Syntaxin 6**						
		NM_001072375	3e-16	Zm.3182.1.A1_at	-0.08	0.52	0.95
**U16**	**r40c1 protein**						
		BT042893	0.00	Zm.886.4.S1_x_at	-0.03	0.90	1.00
**U9**	**Mitochondrial ATP synthase precursor**						
		EU966003	0.00	Zm.5566.1.A1_a_at	0.17	0.10	0.61
		n.d.	0.00	Zm.886.5.S1_a_at	-0.01	0.98	1.00
		n.d.	0.00	Zm.886.5.A1_at	-0.21	0.37	0.90
		n.d.	0.00	Zm.886.2.S1_at	0.14	0.32	0.87
		n.d.	3e-98	Zm.12295.2.S1_a_at	0.21	0.52	0.95
		n.d.	6e-81	Zm.886.1.A1_a_at	0.17	0.81	0.99
**D15**	**Cupin domain containing protein**						
		BT024037	7e-04	Zm.2927.1.A1_at	0.36	0.02	0.30

The putative encoding genes were search by TBLASTN algorithm [GB#, GenBank accession number of maize RNA sequences; TBLASTN score; n.d., not determined]. The putative Affymetrix probe set represented in the GeneChip were searched by BLASTN algorithm in the NetAffx™ Analysis Center data set from Affymetrix (http://www.affymetrix.com/analysis/index.affx). The hybridization and significance values of microarray experiments for these genes are annotated [log_2_(R), p-value and FDR]

## Discussion

Our aim was to identify new elements involved in cellular responses to genomic damage in plants, using CPT as a toxic agent and applying transcriptomic and proteomic approaches to identify the genes, proteins and cellular mechanisms involved. We identified a series of genes and proteins whose expression/accumulation significantly change in response to CPT, although the identified genes do not correspond to the identified proteins. These differences may be a consequence of the different sensitivity of the methods. Moreover, the level of protein accumulation does not necessarily agree with the level of mRNA expression. This incongruent expression between mRNAs and proteins has been observed by other groups, in other species and experimental conditions [78-80] and is most likely a result of the biology of gene expression which includes various levels of regulation during protein synthesis: post-transcriptional, translational, and post-translational. Thus, integrated analysis of both mRNAs and proteins is crucial to gain further insights into complex biological systems.

The basic mechanism of action for CPT has been well-studied and characterised in animal cells [24]. CPT generates replication-mediated DSBs in DNA which in turn induce DNA repair, cell cycle arrest and, under certain circumstances, cell death. Under our conditions, CPT did not induce extensive cell death in maize embryos, as demonstrated by TUNEL staining which only appeared in some cells in the embryo axis after CPT-treatment. At the developmental stage analyzed here, cells in the scutellum divide at a very limited rate, but cells in the embryo axis divide rapidly. This difference may explain the higher sensitivity to CPT of the cells in the embryo axis.

Two basic mechanisms of DSBs DNA repair have been described: homologous recombination and non-homologous end joining [4]. Our transcriptomic analysis identified the induction of some genes already known to be involved in DNA repair. Interestingly, most of them are involved in the HR repair pathway, suggesting that this is the main mechanism for DSBs repair in maize embryos, at least in response to CPT. CPT also produces an increase of a 32 kDa calcium-dependent nuclease activity. However, this nuclease is unlikely to be involved in the extensive fragmentation of the genomic DNA observed in different cell death processes as extensive DNA fragmentation was not observed. Nucleases are also involved in most DNA repair mechanisms, including HR [81]. These data suggests that the 32 kDa nuclease activity observed may be involved in the DNA repair process.

CPT induces reversible or permanent cell-cycle arrest in G2-M phase in human and other cells [82] and produces major alterations in the expression of cell-cycle regulatory genes [83]. We found that CPT reduces the expression of several mitosis-related genes. In addition, we observed a reduction in the accumulation of the histone H2B involved in the structure of chromatin, and changes in the accumulation of two eukaryotic translation initiation factors which seem to also be involved in the cell-cycle process [84]. These results suggest that, in maize embryos, one of the cellular responses to CPT is the arrest of cell division.

In addition to more specific processes, DNA damage induces general stress mechanisms in maize embryos. For example, we observed changes in the expression and accumulation of proteins involved in ROS processing (glutathione S-transferase, Class III peroxidase precursor, chloroplast Cu/Zn superoxide dismutase, cytosolic ascorbate peroxidase), enzymes involved in glycolic metabolism (glyceraldehyde-3-phosphate dehydrogenase, triosephosphate isomerase 1) and in pathogen responses (pathogenesis-related protein1 and Bet v I allergen). Pathogen resistance is increased after DNA damage induction, indicating a cross-link in DNA damage (and maybe other abiotic stresses) and defense responses [85].

An increasing number of studies combining proteomics and transcriptomics clearly demonstrate that mRNA and protein accumulation are not always correlated [86-88]. For instance, in yeast 73% of the variance in protein abundance is explained by the translation mechanism and only 27% due to variations in mRNA concentration [89]. Protein abundance is influenced by several factors at the post-transcriptional, translational, and post-translational levels. For example, there is a time lag between transcription and translation in which introns are excised and the transcripts are moved from the nucleus to the cytoplasm, and translation rates may be influenced by ribosome, tRNA and amino acids availability, codon usage or accessory protein binding association [90]. In addition, protein abundance is also influenced by post-translational processes such as glycosylation, phosphorylation and proteolytic processing.

Our proteome analysis indicated differences in the abundance (up and down) of the encoded proteins of 24 genes whose mRNA levels do not significantly change in response to CPT (Table 4). It is possible that CPT induces the transcription of some genes only during the first hours of treatment, and after three days of treatment the mRNA levels are similar to the control but the abundance of the encoded protein is higher. Differences in the translation rate may also explain the lack of correlation. In animals, post-transcriptional regulation of gene expression during the stress response means specific stress-induced transcripts receive the highest transcriptional priority [91]. Interestingly, some of the spots identified in the proteomic analysis correspond to proteins associated with RNA metabolism and RNA binding proteins, and may be involved in the regulation of mRNA translation.

Many post-translational processes affect the position of a protein in 2D gels such that the protein appears as differentially accumulated in a proteomic analysis. We have identified changes in genes and proteins involved in protein modification and post-translational regulation. For example, the accumulation of at least two 26s proteasome regulatory subunits is altered in response to CPT and the expression of the proteasome inhibitor-like protein PI31 is increased. Ubiquitin/proteasome-mediated protein degradation plays a central role in the regulation of several aspects of plant development and stress responses [92] and our data indicate that it may also be involved in regulating DNA damage responses. In fact, there are evidences that CPT-TOPI-DNA complexes may be degraded by the ubiquitin-dependent pathway in mammals and yeast [27,93]. Our data suggest that a similar situation may occur in plants. Moreover, the expression of *embryonic flower 2 *is repressed, a gene encoding a protein homologous to *Drosophila *Polycomb genes which mediate the epigenetic control of homeotic gene expression [94].

The role of several of the genes identified in the transcriptomic analysis is unknown. These genes may play a role in DNA damage detection and repair mechanisms, especially those genes that are only induced in response to genomic damage and not in response to other types of stress. Unfortunately the data currently available in maize does not allow us to determine which of them are specifically induced by DNA damage, but many of the maize identified genes have clear homologues in Arabidopsis (Table 1 and 2). Microarray analyses in Arabidopsis have been used to study the effects of several abiotic stresses, including two DNA damage agents, bleomycin [4] and gamma radiation [15]. Examining microarray databases [95] we identified eight Arabidopsis genes homologous to maize CPT-induced genes and exclusively induced by DNA damage: At5g02220, of unknown function; At1g13330, encoding TBP-1 tat binding protein; At5g48720, encoding an X-ray induced gene required for post-meiotic stages of pollen development and for male and female meiosis; At3g27060 and At2g21790, encoding the ribonucleotide reductase (RNR) small and large subunit, respectively; At5g20850, encoding AtRAD51; and two genes, At5g18270 and At3g04060, encoding NAC transcriptions factors. NAC proteins constitute one of the largest families of plant-specific transcription factors, and the family is present in a wide range of land plants [96]. These two NAC proteins are interesting candidates for a regulatory role in DNA damage responses in plants.

## Conclusions

The integration of microarray and proteomic analyses provides new data on DNA damage responses in plants. This is a complex process involving DNA repair and arrest of cell-cycle, but also general stress responses. Post-translational processing and the regulation of mRNA translation seem to have an important role in DNA damage responses.

## Methods

### Plant material and treatments

Maize (*Zea mays *L cv W64A pure inbred line) was grown under controlled conditions (16 h light, 28°C). Immature embryos (15 days after pollination) were extracted in sterile conditions and placed on MS plates (4.4% (w/v) Murashige and Skoog medium, 0.8% (w/v) Gelrite) supplemented (or not) with 50 μM camptothecin (Sigma-Aldrich) and maintained in a growth chamber at 26°C in darkness.

### Histological analysis

Embryos were collected, fixed in ethanol-formaldehyde-acetic acid (80:3.5:5) for 1 h at room temperature, followed by 1 week at 4°C, and then stored in 70% ethanol at 4°C. Fixed samples were embedded in paraplast, de-waxed with Histo-Clear II (National Diagnostic, UK), re-hydrated in an ethanol series and equilibrated in 0.02 M citric acid-0.16 M Na_2_HPO_4_, pH 7.0. TUNEL assays were done using the In Situ Cell Death Detection kit (Roche) according to the supplier's protocol for difficult tissues. In negative controls, the TdT enzyme was omitted, and the positive controls were treated with DNase I for 10 min. Experiments were repeated three times.

### RNA extraction and quantification

Total RNA was isolated from frozen samples using the lithium chloride method. DNase digestion of contaminating DNA in the RNA samples was done using RNase-Free DNaseI. Final RNA purification was performed using the RNeasy Mini Kit (Qiagen) according to standard protocols. RNA was quantified with a NanoDrop ND-100 spectrophotometer (NanoDrop Technologies). RNA quality was assessed with a 2100 Bioanalyzer from Agilent Technologies.

### Affymetrix GeneChip hybridization/Microarray analysis

Gene expression was analyzed using the Affymetrix GeneChip^® ^Maize Genome Array, which contains probe sets to interrogate 13,339 genes, performing four independent biological replicates. cDNA synthesis, probe labeling, array hybridization and data analysis were as described by Bannenberg and col. [97], in the Genomics Service of the Centro Nacional de Biotecnologia (CNB-CSIC, Madrid). Raw data and normalised data were deposited at the ArrayExpress data library (http://www.ebi.ac.uk/arrayexpress/) under accession number E-MEXP-2702. Differential expression was considered following the *p *< 0.05 and 2.0 fold change as the criteria of significance. Functional categories of the genes were determined based on Gene Ontology data. We used the Fisher's Exact Test (p ≤ 0.05) and ANOVA (p ≤ 0.01) to determine the significant differences in the functional categories among up- and down-regulated genes.

### Real time quantitative RT-PCR

To validate the expression changes found in the microarray experiments, transcript levels of the ten selected genes were quantified by the ABI Prism 7700 (Applied Biosystems, Foster City, CA, U.S.A) as described by Mascarell-Creus and col. [98]. The oligonucleotides chosen to amplify the selected genes were designed using the Primer Express Software (Applied Biosystems) and are listed in table 5. The actin gene was used as the internal control.

**Table 5:** Sequences of primers used in the real-time PCR experiments.

Name	Sequence
PDIfwd	5'-AAGGGAGACAAGGTACCCATCTC-3'
PDIrev	5'-AAAGACCAACATACTTCCCCTCAAG-3'
LRRfwd	5'-AGGATGCCTGACTGAACTAATGC-3'
LRRrev	5'-GTAAGGTCTCCAACCTCAACATTGT-3'
HRPGfwd	5'-GCCGCGCACGTCATCT-3'
HRPGrev	5'-CGAGCAAACCGTCCAGTAGAC-3'
TBPIPfwd	5'-CGAAGTCCAAGAAATGGAAGAGAA-3'
TBPIPrev	5'-GTAACACCACTCCGCAACTTATTTAG-3'
RNR1bfwd	5'-ATCAAGTTCACAGTGGATACC-3'
RNR1brev	5'-TCTAGCTTCCACACGCC-3'
RAD51fwd	5'-GTTTGGCTTGAATGGCG-3'
RAD51rev	5'-TAAAGGGCTGTAGCACTAT-3'
XRI-1fwd	5'-CGTCACAACCGCAAATG-3'
XRI-1rev	5'-CGTACAAGATCCGGCAC-3'
CycBfwd	5'-AGGCAGAGCTCATTGAATGCA-3'
CycBrev	5'-CAGTCGAGTGCGCGATCA-3'
B3fwd	5'-AGGCAGAGCTCATTGAATGCA-3'
B3rev	5'-GATTCAACTGTTTTTCAGTTGATTAAGC-3'
E98fwd	5'-ACCTTCTGTCATGTTCTTCCATTCT-3'
E98rev	5'-GAATGTACCTGAGATTGGCAAGATC-3'

The oligonucleotides were designed using the Primer Express software (Applied Biosystems).

### In-gel Nuclease Activity Assay

Immature maize embryos were ground in liquid nitrogen and resuspended in extraction buffer (150 mM Tris-HCl, pH 6.8, 0.5 mM PMSF, 20 μM leupeptin). The homogenate was clarified by centrifugation at 12,000 g for 5 min at 4°C. In-gel nuclease activity was measured according to Thelen and Northcote [99] using 10 μg of protein in 12.5% SDS-PAGE containing 50 μg/mL of single-stranded calf thymus DNA (Sigma) and 50 μg/mL bovine fibrinogen (Sigma). After electrophoresis, gels were washed twice in 25% (v/v) isopropanol, 10 mM Tris-HCl pH 7.0 for 30 min and twice in 10 mM Tris-HCl, pH 7.5 for 30 min. Gels were incubated overnight at 37°C with gentle agitation in 10 mM Tris-HCl, pH 7.5, 1 mM CaCl_2_, 1 mM MgCl_2 _for Ca^2+^/Mg^2+^-dependent activity or in 25 mM NaAc/HAc pH 5.5, 1, 2 or 5 mM ZnSO_4_, for Zn^2+^-dependent activity. Nuclease activity was detected by staining the gel with 1 μg/mL (w/v) ethidium bromide for 15 min and observed under UV.

### Two-dimensional gel electrophoresis

Maize embryos were ground in liquid nitrogen and crude protein extracts were solubilised in 1.2 ml buffer 1 (7 M urea, 2 M thiourea, 4% CHAPS, 4% Triton X-100, 18 mM Tris-HCl pH 8) in the presence of 53 u/ml DNase I, 4.9 u/ml RNaseA and a cocktail of protease inhibitors (1 mM PMSF, 50 μM leupeptin, 1 μM pepstatin, 10 μM E-64, 10 μg/ml aprotinin). After 20 min incubation at 4°C, DTT at a final concentration of 14 mM was added and samples were centrifuged 10 min at 35000 *g *at 4°C. 2-DE analysis was performed basically as previously described [100] using pH 3-11, 24 cm immobilised pH gradient (IPG) strips (Immobiline DryStrips, GE Healthcare) for the first dimension. The optimal parameters for spot detection were: smooth = 4, saliency = 1.0 and minimum area = 5. To evaluate protein expression differences among gels, relative spot volume (% Vol.) was used. Protein abundance variation was validated by Student's t-Test (p < 0.05).

### In-gel digestion of proteins and MS and MS/MS spectra

Proteins were in-gel digested with trypsin and tryptic peptides were extracted and analyzed by MALDI-TOF/MS (4700 Proteomics Analyzer, Applied Biosystems) or LC-ESI-QTOF (Q-TOF Global, Micromass-Waters) mass spectrometers in the Proteomics Platform (PCB) of the University of Barcelona as previously described [100].

## List of Abbreviations

CPT: camptothecin; TOPI: DNA topoisomerase I; DSBs: double strand breaks; RNR: ribonucleotide reductase; PCD: programmed cell death; TUNEL: *in situ *detection of fragmented DNA; MS/MS: tandem mass spectrometry; FDR: false discovery rate; HR: homologous recombination.

## Authors' contributions

NSP and CV designed and performed most of the experiments; NSP and SI performed the proteome experiments, SI analyzed the proteomics data. NG designed and analyzed the results of the qRT-PCR analyses. NSP and CV analyzed the data and wrote the manuscript. All authors read and approved the manuscript.
